# Alleviating the unwanted effects of oxidative stress on Aβ clearance: a review of related concepts and strategies for the development of computational modelling

**DOI:** 10.1186/s40035-023-00344-2

**Published:** 2023-03-13

**Authors:** Sarawoot Somin, Don Kulasiri, Sandhya Samarasinghe

**Affiliations:** 1 Centre for Advanced Computational Solutions (C-fACS), Lincoln University, Christchurch, 7647 New Zealand; 2 Department of Wine, Food and Molecular Biosciences, Lincoln University, Christchurch, 7647 New Zealand; 3 Centre for Advanced Computational Solutions (C-fACS), Lincoln University, Christchurch, 7647 New Zealand

**Keywords:** Alzheimer’s disease, Oxidative stress, Amyloid beta clearance, Protein design

## Abstract

Treatment for Alzheimer’s disease (AD) can be more effective in the early stages. Although we do not completely understand the aetiology of the early stages of AD, potential pathological factors (amyloid beta [Aβ] and tau) and other co-factors have been identified as causes of AD, which may indicate some of the mechanism at work in the early stages of AD. Today, one of the primary techniques used to help delay or prevent AD in the early stages involves alleviating the unwanted effects of oxidative stress on Aβ clearance. 4-Hydroxynonenal (HNE), a product of lipid peroxidation caused by oxidative stress, plays a key role in the adduction of the degrading proteases. This HNE employs a mechanism which decreases catalytic activity. This process ultimately impairs Aβ clearance. The degradation of HNE-modified proteins helps to alleviate the unwanted effects of oxidative stress. Having a clear understanding of the mechanisms associated with the degradation of the HNE-modified proteins is essential for the development of strategies and for alleviating the unwanted effects of oxidative stress. The strategies which could be employed to decrease the effects of oxidative stress include enhancing antioxidant activity, as well as the use of nanozymes and/or specific inhibitors. One area which shows promise in reducing oxidative stress is protein design. However, more research is needed to improve the effectiveness and accuracy of this technique. This paper discusses the interplay of potential pathological factors and AD. In particular, it focuses on the effect of oxidative stress on the expression of the Aβ-degrading proteases through adduction of the degrading proteases caused by HNE. The paper also elucidates other strategies that can be used to alleviate the unwanted effects of oxidative stress on Aβ clearance. To improve the effectiveness and accuracy of protein design, we explain the application of quantum mechanical/molecular mechanical approach.

## Introduction

The high mortality rate due to Alzheimer’s disease (AD), and the high costs associated with AD patient care, have become global issues of concern, both for the individuals it directly affects and for those in charge of their care [1, 2]. The extracellular accumulation of aggregated amyloid beta (Aβ) and intracellular tau-containing neurofibrillary tangles found in hippocampus and cerebral vasculature (including the neocortex) [3], have been identified as potential pathological hallmarks of neuronal dysfunction that results in AD. Aβ is produced as a result of cellular metabolism in healthy neurons [4]. BACE1, known as the beta-secretase cleaving precursor protein implicated in AD, has also been identified to play a role in axon guidance of olfactory sensory neurons in the olfactory bulb [5, 6]. An imbalance between Aβ aggregation and clearance leads to increased toxicity [7]. Thus, maintaining a balance between Aβ aggregation and clearance may provide a viable therapy for AD [8]. To alleviate the progress of AD, treatment should begin in the early stages of the disease [9]. Since initial Aβ aggregation occurs in the early stages of AD [10], understanding the pathways associated with Aβ aggregation, which has been linked with AD, may offer a form of therapeutic intervention [11].

Although both genetic mutations and non-genetic factors result in Aβ accumulation [12], in terms of Aβ accumulation, non-genetic reasons may be more explicit than genetic ones [11, 13]. Oxidative stress, a well-known non-genetic reason for early-stage AD, occurs when there is an imbalance between antioxidant defences and the productions of free radicals [14]. This imbalance leads to the progression and pathogenesis of AD in the early stage, by reducing Aβ clearance [15]. Many studies have shown that oxidative stress is involved in other neurodegenerative diseases such as Parkinson’s disease, ischemic stroke, diabetes mellitus, and cancer [16, 17]. 4-Hydroxynonenal (HNE), the product of lipid peroxidation, results from oxidative stress [18]. HNE is abundant when there are high levels of ROS toxicity [19]. This toxicity may lead to a reduction in the structural modification of proteases [20]. Subsequently, Aβ-degrading proteases display a decrease in the expression of enzyme activities, in particular, Aβ clearance [21, 22]. Since the degradation of HNE-modified protein may enhance Aβ clearance, it is crucial to understand how HNE interacts with and modifies proteins.

In general, HNE-modified proteins, defined as aberrant cellular components, are degraded via the autophagy-lysosome pathway and the ubiquitin–proteasome pathway (UPP) [23]. Another activity which protects against oxidative stress is antioxidant activity: it restrains oxidative chain reactions. Techniques to enhance the antioxidant activity may reduce oxidative stress; for example, regulating the expression of glutathione (GSH), the most prevalent antioxidants in the brain cells, resists oxidative stress [24, 25]. Nanozymes, which mimic the expression of the antioxidants, have recently gained popularity due to their low development costs [26]; this technology is based on protein design [27]. Another well-known factor involved in oxidative stress is insulin resistance [28]. As there are many factors that cause oxidative stress, the reduction of such stress still presents challenges for therapeutic intervention [29].

In this review, we discuss neurobiological pathways associated with AD and the interplay of pathological factors which contribute to the disease. We also investigate the generation of oxidative stress which results in the impairment of Aβ clearance, including the degradation of the HNE-modified protein and the mechanisms associated with the antioxidants. We identify techniques that could alleviate the unwanted effects of oxidative stress, focusing on those that mimic antioxidant activity, the desirable function of protein inhibitors, and related concepts of protein design.

## Neurobiological pathways associated with AD and other mechanistic aspects of AD

### Neurobiological pathways

**Fig. 1:**
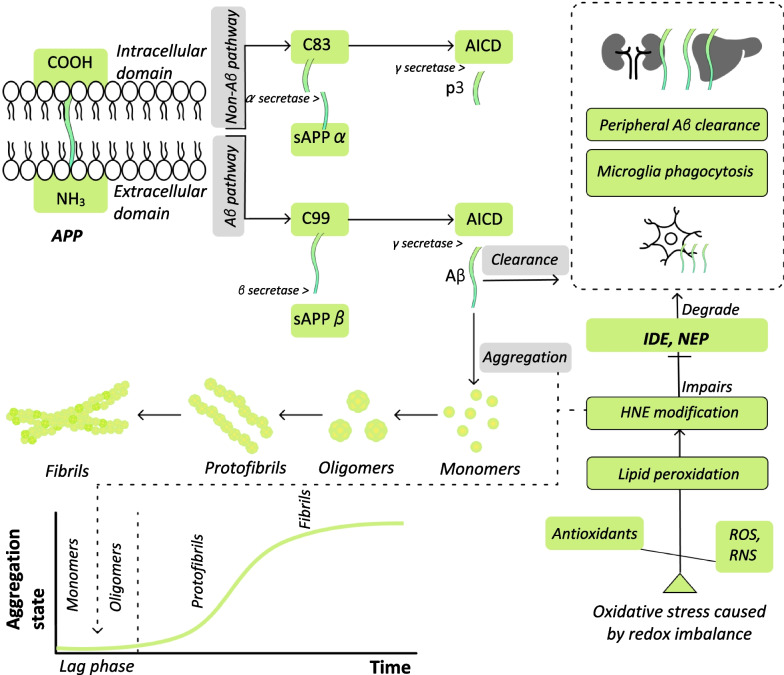
Schematic presentation of proteolytic processing of the amyloid precursor protein (APP), the Aβ clearance mechanism, and the Aβ aggregation process which occurs via HNE modification. The proteolytic processing of the APP could be divided into two pathways: non-amyloidogenic and amyloidogenic. In the non-amyloidogenic pathway, APP is cleaved by α-secretase which results in the production of C83 and sAPPα. Ultimately, γ-secretase cleaves C83 and p3 is produced, which precludes Aβ aggregation. For the amyloidogenic pathway, APP is cleaved by β-secretase which results in the production of C99 and sAPPβ. Subsequently, the γ secretase cleaves C99 and produces AICD and Aβ peptides. Initial Aβ aggregation is considered as a hallmark pathology for diagnosing AD in the early stage (lag phase). In Aβ clearance, Aβ peptides are degraded via IDE and NEP proteases through microglial phagocytosis and peripheral Aβ clearance. However, oxidative stress can impair Aβ clearance through 4-hydroxynonenal (HNE) modification; HNE modification is the product of oxidative stress due to the presence of lipid peroxidation in the lag phase of AD. Oxidative stress reflects a redox imbalance, resulting from a combination of reactive oxygen species (ROS) and reactive nitrogen species (RNS) overweighing the antioxidants

Enzymatic processing of amyloid precursor protein (APP) results in production of several derivatives with biological functions. Toxic products of APP are known as the factors involved in the pathology of AD. APP proteins, produced by the endoplasmic reticulum, are transported to the Golgi complex and ultimately to the plasma membrane. APP cleavage by β-secretase between positions 16 and 17 produces the β-C-terminal fragment (C99) and the large ectodomain (sAPPβ), through the amyloidogenic pathway. This process ultimately enhances Aβ aggregation. APP cleavage by α-secretase between positions 10 and 11 produces the α-C-terminal fragment (C83) and the ectodomain (sAPPα) through the non-amyloidogenic pathway, which prevents Aβ aggregation [30, 31]. C99 and C83 are then cleaved by γ-secretase to produce Aβ and p3 peptides through amyloidogenic and non-amyloidogenic pathways, respectively (Fig. 1). In addition to the amyloidogenic pathway, C99 can be cleaved by α-secretase to produce other Aβ species [32]. Previous studies have identified that many cleavages of APP, including short Aβ isoforms (Aβ1-17/18/19/20), are produced through the amyloidogenic pathway [33–35]. Although the mechanisms and functions of APP are not completely understood, studies have shown that the production of APP is related to transcriptional control, axonal transport, and apoptosis [36–38].

Aβ monomer, known as Aβ protein, has two dominant forms of Aβ proteins, Aβ40 and Aβ42. Evidence indicates that the Aβ monomer may be non-toxic [39, 40]. Aβ monomers may aggregate into an aggregation state, resulting in Aβ oligomers (AβOs) and protofibrils, which have low and high molecular weights, respectively. These protofibrils ultimately form fibrils and amyloid plaques [41]. Researchers have reviewed the relationship between the products of the amyloidogenic pathway using in vitro, in vivo, and in silico (computer simulation) experiments [42, 43]. Pharmacological experiments have also shown the relationship between the products of the amyloidogenic pathway needed for the development of pharmacological interventions [42, 43].

### Aβ oligomers

Although researchers once thought that amyloid plaque was associated with Aβ aggregation or a pathogenic form of Aβ, they have now identified AβOs as the pathogenic form of Aβ, as indicated by the AβOs present in animal and human models [44–46]. For instance, low levels of Aβ but not AβOs are present in severe cognitive impairments like AD [47, 48].

Studies have identified AβOs as contributors to poor memory [49]. Experimental studies in cell biology have proven this: non-transgenic mice injected with small quantities of AβOs show poor memory performance [50, 51]. Likewise, in vivo studies have shown that AβOs disrupt long-term potentiation and contribute to long-term depression [52]. AβOs may be more cytotoxic to neuronal synapses than protofibrils and fibrils based on experimental results of in vivo studies [53–55]. To understand oligomer production and toxicity, researchers have investigated the monomer-dependent secondary nucleation (MDSN) of Aβ in an in vitro study [56]. They found that the rate of the MDSN process plays a key role in amyloid-forming peptides; knowing the rate of the MDSN process, modulated by hydrophobic and electrostatic interaction of surrounding proteins, will ultimately help in the development of inhibitors which suppress MDSN.

### Other mechanistic aspects of AD

Researchers have investigated other mechanistic aspects related to Aβ toxicity. For example, researchers have found that mutation of insulin-degrading enzyme (IDE) at the allosteric site, resulting in the cysteine-free IDE mutant which is catalytically inactive against insulin, impairs Aβ degradation [57]. This is a known cause of Aβ deposition and toxicity. While the deposition of functional toxic forms of Aβ in the central nervous system can cause AD, non-functional toxic forms of Aβ deposited in the tissue lead to other diseases such as amyotrophic lateral sclerosis (ALS) [58]. Researchers have explored Aβ toxicity in AD using in vitro experiments and by disrupting membranes [59]. The latter has demonstrated that cell membrane disruption is comprised of two steps: soluble AβOs binding to the membrane, and Aβ fibrils causing membrane fragmentation. In the first step, the soluble AβOs bind to the membrane to form calcium-permeable pores, and they are known as primary pathologic species of AD. In the second step, elongated Aβ fibrils, as the detergent molecules, interact with cell membranes, causing membrane fragmentation by detergent effect [59].

### Conformation of AβOs, transformed by other agents

In the lag phase or early stages of AD, the conformation of AβOs may be dominated or changed by numerous toxic pathways. These pathways include various agents such as metal, other amyloid proteins (such as synuclein and tau), and lipids. For instance, aberrant metal homeostasis may dominate the conformation of AβOs. Zinc, copper, and iron may mediate the aggregation of AβOs [60]. These metals may react with the products of free radicals, leading to cellular toxicity [61]. Studies have identified that pre-formed AβOs, which interact with lipids as well as Aβ monomers, can affect the conformation of AβOs [62, 63]. Regarding the toxicity of AβOs, the interaction between AβOs and the lipid membrane has been identified as a prominent factor in neuronal cell damage [63]. Interactions between other amyloid proteins and AβOs have been explored to understand different amyloid diseases [64]. For example, prion protein, which can cause fatal diseases, may be a high-affinity receptor for AβOs leading to conformational change and aggregation of AβOs [65]. This phenomenon, or the links between other amyloid proteins (misfolded proteins) and AβOs (which are based on molecular and pathogenic mechanisms), are known as cross-seeding. Cross-seeding interrupts the conformation of AβOs and Aβ aggregation. Recent studies have investigated the cross-seeded polymerisation of Aβ to understand the underlying mechanism of amyloid-forming proteins [66]. Examinations of cross-seeding have provided crucial information for those developing therapeutic interventions for AD, using the mechanisms of related diseases [67, 68].

### Development of antibodies against AβO toxicity

Immunotherapies using antibodies against AβO toxicity are known as one of the most promising approaches for pharmacological interventions of AD. Injection of an AβO-specific antibody has been shown to rescue memory performance (spatial learning) in transgenic mice (5×FAD mice) [69]. Kinetic analyses have shown that antibodies protect cell membranes against AβO toxicity [70, 71]. Using high-throughput screening technology, studies have shown that 5 small molecules can inhibit or prevent AβO toxicity [72]. An antibody-based immunotherapeutic approach has been applied to inhibit activities of soluble oligomers and insoluble fibrils [73]. The antibody-based immunotherapeutic approach reported by Sevigny et al. selects human B-cell clones triggered by the unique antigens (neo-epitopes) present in pathological Aβ aggregates during the process of Aβ aggregation [73].

Scientists have also developed antibodies to reduce or counteract the effects of certain metals. Various metals contribute to AβO toxicity and senile plaques, which can increase concentrations of transition metals in AD-affected brains [74, 75]. However, the development of drugs and nonpharmacological procedures to slow down or halt AD has been hindered by the fact that the complex heterogeneous properties of AβOs are not completely understood, particularly from a mechanistical perspective [76, 77].

## The effect of oxidative stress on Aβ-degrading proteases

There are many processes involved in Aβ homeostasis, including its deposition into insoluble aggregates, the active transport out of the brain, proteolytic degradation, and cell-mediated clearance [30]. A study of Aβ-associated pathology found that proteolytic degradation is an important determinant of Aβ aggregation [78]. Aβ-degrading proteases, known as particularly important biomolecules of the immune system found in the brain, play a central role in the process of proteolytic degradation which enables Aβ clearance [79].

### Mechanisms of Aβ proteases

There are approximately 20 Aβ-degrading proteases (both intracellular and extracellular) involved in Aβ clearance. These proteases include the IDE, neprilysin (NEP), endothelin-converting enzyme 1 and endothelin-converting enzyme 2 [80]. Aβ-degrading proteases, produced by glial cells, cleave Aβ peptides into smaller fragments at different sites. An in silico study has shown that the Aβ-degrading proteases possess many cleavage sites in the Aβ peptide [81]. Up-regulation of these proteases helps to control the aggregation of Aβ peptides and thus presents a possible avenue for therapeutic intervention [81]. Scientists have recently investigated the characteristics of peptide fragments degraded by the prominent proteases –NEP and IDE [79].

### Other mechanisms of Aβ clearance

Another crucial process for Aβ clearance involves the active transport of these proteins from the brain. Many proteins play a crucial role in Aβ clearance, including apolipoprotein E (APOE) and α2-macroglobulin (α2-m) [82]. APOE and α2-m interact with various receptors, including lipoprotein receptors. As a result of these interactions, small fragments or Aβ sequences cross the blood–brain barrier (BBB) [83]. In short, the level of Aβ clearance may not only be determined by proteolytic degradation, but also by whether it is actively transported out of the brain across the BBB.

Both genetic and non-genetic factors can cause an increase in the aggregation of Aβ peptides and lead to Aβ catabolism [22, 81]. Researchers have identified genetic mutations and non-genetic factors as causes of familial AD and sporadic AD (SAD), respectively. Non-genetic SAD account for 90% of all AD cases [84]. In addition, non-genetic factors play a greater role than genetic factors in the impairment of Aβ clearance [79].

In amnestic mild cognitive impairment (aMCI), or early-stage AD, several non-genetic factors may affect the metabolism of glucose, leading to mild cognitive impairment [28]. Intervention is crucial at this stage to ensure aMCI individuals can continue to function well [85]. In aMCI, oxidative stress is one non-genetic factor associated with impaired Aβ clearance and enhanced Aβ aggregation via HNE modification [11, 86]. Also, oxidative stress resulting in impaired Aβ clearance and enhanced Aβ aggregation is considered as an initial lag phase [86] (Fig. 1)**.**

### Oxidative stress and the production of HNE

Oxidative stress is an imbalance between free radicals and antioxidants, where the number of free radicals outweighs the number of antioxidants. Free radicals are oxygen-containing molecules with an uneven number of electrons, which enables them to interact with other molecules known as oxidants or reductants [87]. Free radicals like hydroxyl radicals, hydrogen peroxide, superoxide anion radicals, hypochlorite, nitric oxide radicals, and peroxynitrite radicals are believed to cause neurological diseases such as Parkinson’s disease and AD [88]. In the context of chronic oxidative stress, these free radicals negatively impact certain processes, leading to oxidative protein modification, DNA oxidation, and lipid peroxidation of the cell membrane [89, 90]. These processes eventually result in homeostatic disruption and cell damage [87]. In normal conditions, antioxidants neutralise free radicals by donating electrons to them. As a result, free radicals become less reactive and more stable [91]. The balance between the production of free radicals and the antioxidant activity is indicated by redox signalling [14, 92]. The redox status has been explored in neurodegenerative diseases such as AD and Parkinson’s disease [93, 94].

The covalent modification of aldehydes by lipid peroxidation, known as oxidative carbonylation, will ultimately lead to oxidative protein modification. This plays a key role in metabolic diseases [20]. Oxidative carbonylation means that free radicals directly attack specific amino acids which are vulnerable to oxidation (e.g., proline, arginine, lysine, and threonine), leading to protein hydrophobicity (protein unfolding) and the risk of protein aggregation [95, 96]. Likewise, the free radicals oxidise DNA bases (adenine, guanine, cytosine, and thymine), leading to DNA damage. For example, guanine, which has high oxidation potential [97, 98], is attacked by free radicals at its imidazole ring. As a result, guanine is transformed into 8-hydroxyguanine, causing DNA lesions [99]. Lipid peroxidation is known as a prominent source of cell membrane damage [90]. Lipid peroxidation of the cell membrane occurs when free radicals, under oxidative stress conditions, attack the cell membrane at the carbon–carbon double bond(s), causing hydrogen removal from carbons and oxygen insertion. A lipid peroxyl radical is formed and an abstract hydrogen atom, in fatty acyl chain in a lipid bilayer, forms lipid hydroperoxide (LOOH) [100]. In the propagation phase, the free radicals can react with the lipid peroxyl radicals, by removing hydrogens from the lipid molecule, resulting in the production of new free radicals and lipids [101, 102]. Many studies have found that lipid peroxidation contributes to the development of pathological states and accelerates aging [20, 103, 104]. The brain is vulnerable to attacks by free radicals because the phospholipid form, which is the backbone of neuron membranes, contains high levels of polyunsaturated fatty acids (PUFAs). PUFAs, like glycolipids, phospholipids, and cholesterol, are a family of lipids with carbon–carbon double bonds [90, 105]. In addition to LOOH, lipid peroxidation can produce many toxic secondary products, of which HNE is the most toxic one [90, 106]. HNE, when bound to the key neuronal membrane, results in dysfunction of key neuronal proteins, leading to neuronal death. HNE is produced by lipid peroxidation through two pathways: enzymatic and nonenzymatic pathways [90, 107].

**Fig. 2:**
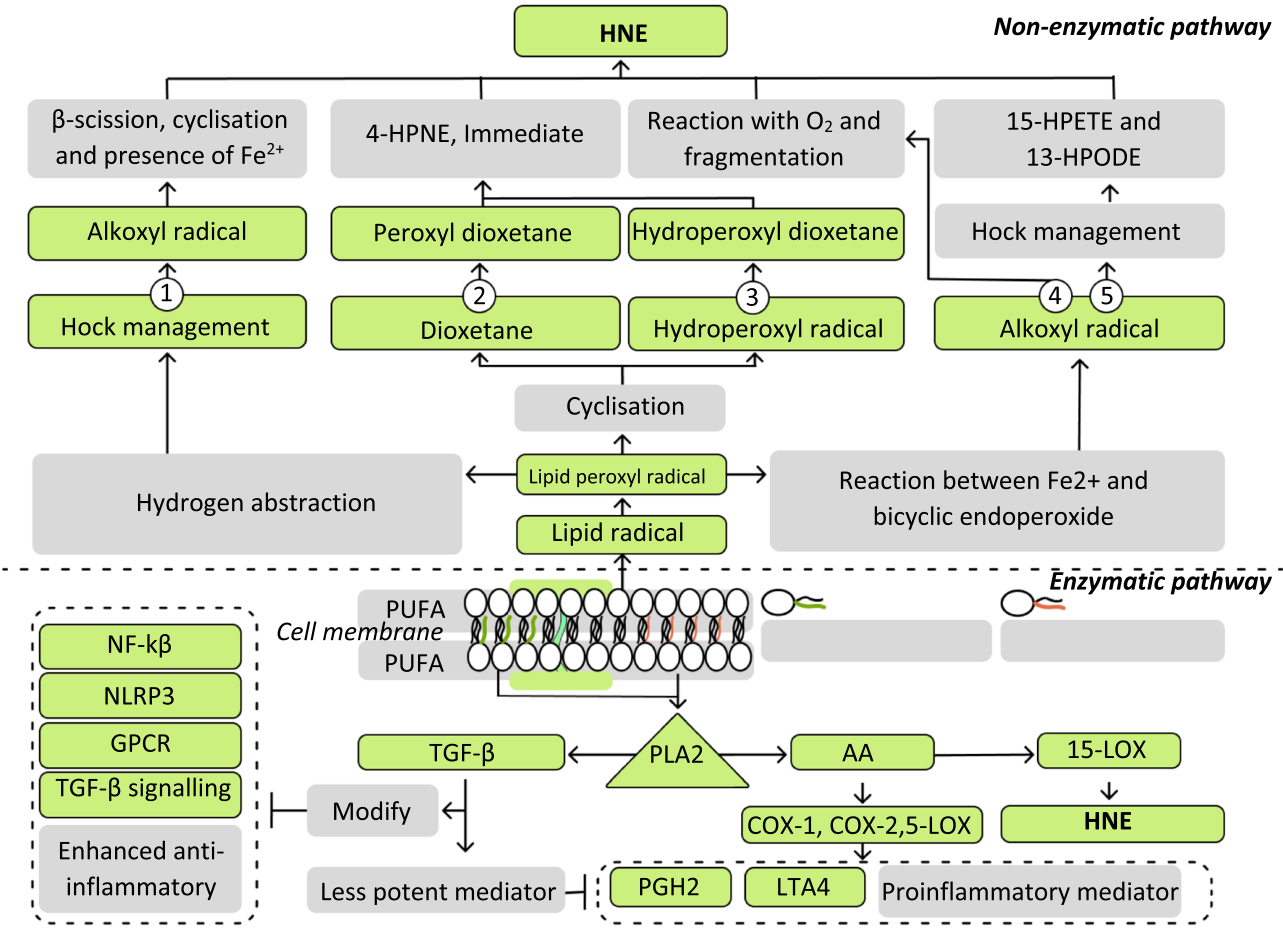
Schematic diagram illustrating two pathways associated with the production of HNE: the enzymatic pathway and the non-enzymatic pathway. In the enzymatic pathway, the PLA2 cleaves n-3 PUFAs and produces EPA and DHA. The cleavage product of the n-6 PUFA family is arachidonic acid (AA). EPA and DHA inhibit NF-kB, NLRP3, GPCR, and TGF-β signalling. AA, produced by PLA2 and catalysed by COX-1, COX-2, and 5-LOX, results in PGH2 and LTB4, leading to the production of proinflammatory mediators. AA is also converted into HNE via 15-LOX. For the non-enzymatic pathway, the free radical lipid peroxidation primarily produces HNE. Abstraction of the allylic hydrogen atom remains lipid radical, resulting in a carbon-centred alkyl radical; eventually, the carbon-centred alkyl radical produces lipid peroxyl radical. The lipid peroxyl radical may generate HNE via five mechanisms. (1) The hydroperoxyl radical is produced as a result of the hydrogen abstraction of the lipid peroxyl radical. The alkoxyl radical produces HNE via β-scission, cyclisation, and the presence of the transition metal ion Fe^2+^. (2) Hydroperoxyl dioxetane, formed through cyclisation, produces peroxyl dioxetane through oxygenation. Peroxyl dioxetane then produces 4-HPNE by fragmentation. Here 4-HPNE becomes HNE by hydrogen abstraction. (3) The hydroperoxyl radical is produced by hydroperoxyl dioxetane through cyclisation. HNE is produced from hydroperoxyl dioxetane as a result of fragmentation and the abstraction of hydrogen. (4) The reaction between Fe^2+^ and bicyclic endoperoxides creates alkoxyl radicals. These alkoxyl radicals cause HNE through oxygenation and fragmentation processes. (5) 15-HPETE and 13-HPODE, produced by the alkoxyl radicals through Hock rearrangement and cleavage, are known as immediate precursors of HNE

In the enzymatic pathway, PUFAs are cleaved by phospholipase A2 (PLA2) at the sn-2 position, a process which frees PUFAs from neuron membranes [108]. There are two major classes of PUFAs: omega-3 PUFAs (n-3 PUFAs) and omega-6 PUFAs (n-6 PUFAs). Both classes are metabolised by the same esterification reaction [109] **(**Fig. 2), and both require release of PUFAs from cell membranes [109]. The n-3 PUFA family comprises α-linolenic acid, docosahexaenoic acid (DHA), and eicosapentaenoic acid (EPA). The n-6 PUFA family includes linoleic acid, arachidonic acid (AA), and dihomo-γ-linolenic acid. While n-3 PUFAs exert anti-inflammatory effects and vasodilation, n-6 PUFAs cause inflammation and platelet aggregation. The anti-inflammatory activities of n-3 PUFAs are related to the fact that n-3 PUFAs—EPA and DHA—can be enzymatically converted to generate bioactive and anti-inflammatory products. They also exert anti-inflammatory effects through modulating nuclear factor-κB signaling, NLRP3 (NOD-like receptor family pyrin domain containing 3) inflammasome, G protein-coupled receptors, and transforming growth factor β signalling [110]. For n-6 PUFA oxidative metabolism, AA can be converted to prostaglandin H2 (PGH2) by cyclooxygenases 1 and 2 [111]. AA can also be converted to leukotriene B4 (LTB4) by 5-lipoxygenase (5-LOX) and leukotriene A4 (LTA4) hydrolase. Both PGH2 and LTA4 can cause a variety of illnesses [112]. In addition to n-6 PUFA oxidative metabolism, AA can also be transformed into HNE through the metabolism of 15-lipoxygenase (15-LOX) [113]. Of the two, the non-enzymatic lipid peroxidation pathway has received more scientific attention as the non-enzymatic lipid peroxidation of PUFAs leads to the formation of intensively reactive electrophilic aldehydes—HNE, malondialdehyde, and acrolein [114].

In the non-enzymatic pathway, production of HNE is involved in the free radical lipid peroxidation [86]. PUFA first undergoes abstraction of allylic hydrogen atom from the methylene group to produce a carbon-centred alkyl radical. Then, the alkyl radical produces peroxyl radical. As illustrated in Fig. 2 and explained below, HNE is produced via five mechanisms. First, the peroxyl radical forms hydroperoxyl radicals, which in turn, are involved in hydrogen abstraction [115]. Then, the hydroperoxyl radical and the alkoxyl radical produce HNE through transition metal ions—such as Fe^2+^—and beta-scission regulated by a hydroxy alkoxy radical. Second, cyclisation of the peroxyl radical forms dioxetane, followed by oxygenation of dioxetane, which leads to peroxyl dioxetane. This fragmentation of peroxy-dioxetane leads to 4-hydroperoxy-2E-nonenal (4-HPNE); eventually, the hydrogen abstraction of 4-HPNE results in HNE. Third, oxygenation of the hydroperoxyl radical results in hydroperoxyl dioxetane, which is further fragmented to 4-HPNE, the immediate precursor of HNE. Fourth, alkoxyl radicals, produced by a reaction between reduced forms of transition metals and bicyclic endoperoxides, are oxygenated and fragmented, generating HNE. Fifth, the alkoxyl radical is cyclised and oxygenated. It undergoes a Hock rearrangement. This process generates 15-hydroperoxyeicosatetraenoic acid (15-HPETE) or 13-hydroperoxy-linoleic acid (13-HPODE), both of which are known as immediate precursors of HNE.

### Oxidative stress and impairment of Aβ-degrading proteases, via HNE modification

Aβ-degrading proteases are an important feature of the immune system found in the brain and play a key role in Aβ clearance [79]. Understanding how these Aβ-degrading proteases break down the Aβ peptide via proteolysis may help develop targeted AD treatments [116]. One study has found that up-regulation of these proteases helps control the accumulation of Aβ peptides [81]. IDE and NEP are the most significant enzymes involved in Aβ degradation. They are released from microglial cells [79, 117]. Both of these enzymes are known as intracellular and extracellular Aβ-degrading enzymes [22]. Intracellularly, interaction between the NEP and APP intracellular domain (AICD) enables amyloid clearance through the regulation of NEP [118]. Subsequently, ACID may be released into the cytosol to be degraded by IDE [106]. Both NEP and IDE are well-known extracellular degrading enzymes [117, 119, 120].

Aging and high cholesterol levels may cause low level and low activity of NEP and IDE [121, 122]. Furthermore, several studies have shown that oxidative stress, through post-translational modification and lipid peroxidation, can impair the expression of NEP and IDE in AD [123–125]. Protein misfolding, caused by interactions between lipid peroxidation products and proteins, impairs protein activities of NEP and IDE, and ultimately leads to Aβ aggregation [126, 127]. For instance, HNE-NEP adduction can reduce Aβ cleavage, a key factor in Aβ accumulation [127]. Likewise, HNE-IDE adduction may lower the enzymatic activity of IDE [79, 127].

**Fig. 3:**
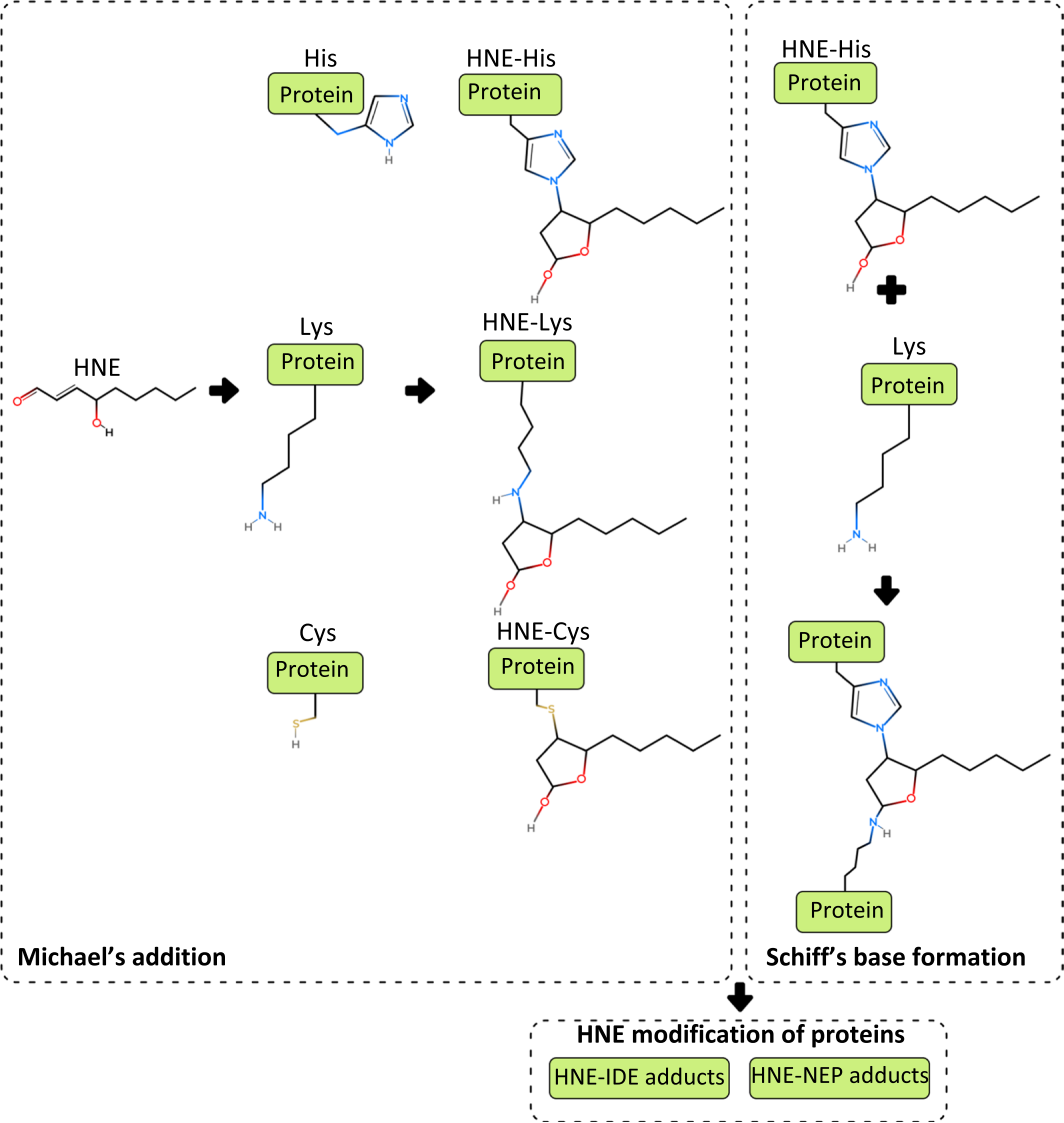
Schematic presentation of HNE modification of amino acids using Michael’s addition concept and Schiff’s base formation concept. HNE is 9-carbon-atom long, with a double bond between carbon atoms 2 and 3 (C2 and C3). These atoms interact with the head aldehyde group at carbon atom 1 (C1) and the hydroxyl group at carbon atom 4 (C4). Carbon atoms 5 through to 9 (C5, C9) are hydrophobic. Michael’s addition to HNE occurs at the double bond (C=C) and carbonyl group (C=O) [136]. Amino acids interacting with HNE at C=C and C=O, consist of His, Cys and Lys. In Schiff’s base formation, HNE-His modification, a product of Michael’s addition, can interact with Lys. As a result of Michael’s addition and Schiff’s base adduction, HNE-IDE and HNE-NEP adducts cause impaired Aβ clearance

The binding of HNE to amino acids may be explained by two principles: Schiff’s base formation and Michael’s addition (Fig. 3). HNE often reacts with lysine (Lys) and histidine (His), amino acids of NEP and IDE; it also reacts with cysteine (Cys) at the same velocity as Lys and His [20, 128, 129]. The interaction between HNE and these amino acids may modify chemical structure of NEP and IDE. For example, the HNE-induced modification of Cys, His and Lys can impair the enzymatic activity of NEP [130]. Similarly, an abundance of Cys and His, modified by HNE, can lower the activity of IDE [131, 132]. One study found that the reactions of HNE with Lys, His, and Cys have the same velocity. Cys has the highest reactivity, followed by His, Lys and Arg [20].

## Degradation of the HNE-modified proteins

Since the accumulation of HNE-modified proteins may be involved in degenerative diseases and cellular aging [133], the degradation of the HNE-modified proteins is a crucial mechanism for cellular and organismal homeostasis. One in vivo study has shown that HNE modification negatively affects the structural and functional dynamics of L-FABP (liver fatty acid-binding protein) [134], impairing the transference of fatty acid through the cell membrane.

The HNE-modified proteins are degraded by natural protein degradation pathways in order to maintain normal cellular function. Meanwhile, researchers have developed defensive strategies to prevent the HNE modification, which protect against oxidative stress, by mimicking natural synergetic antioxidants [135].

### Pathways involved in the degradation of HNE-modified proteins

**Fig. 4:**
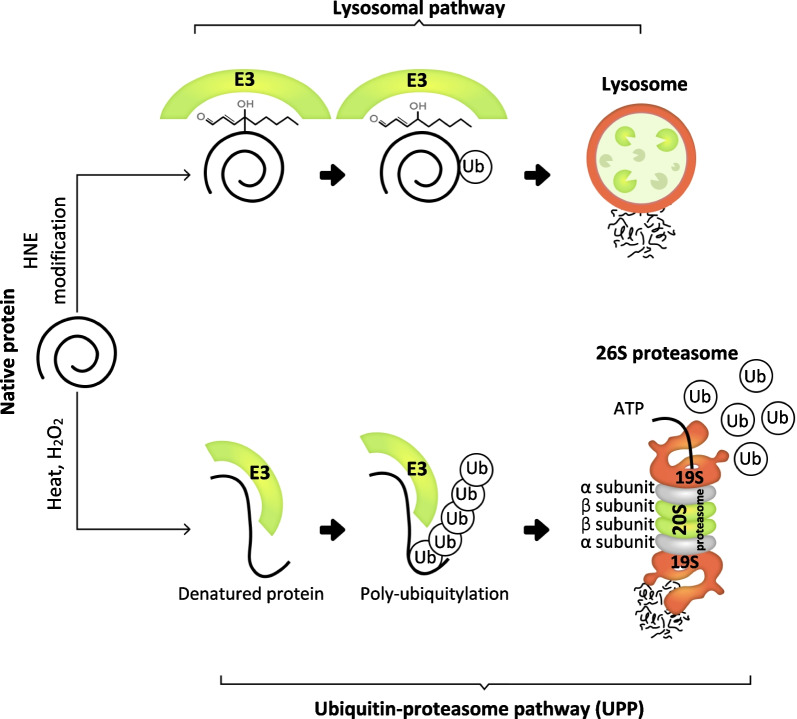
Schematic illustration of the degradation of modified proteins via the UPP and lysosomal pathways. The native protein, denatured by heat or H_2_O_2_, is degraded through the UPP pathway. The HNE-modified protein is degraded through the lysosomal pathway. In the UPP pathway, the ubiquitin ligase (E3) interacts with both the denatured protein and the conjugated enzyme. This process, known as protein ubiquitination modification, enables the lysine residue to interact with the ubiquitin chain (Ub). This protein ubiquitination modification produces a polyubiquitinated protein. E3 transfers the polyubiquitinated protein to the 26S proteasome. Ultimately, the polyubiquitinated protein is conjugated with the 19S proteasome (receptor) and degraded by the 20S proteasome, which contains the cleavage sites at the β subunits [159]. In the lysosomal pathway, E3 interacts with the HNE-modified protein and catalyses the transfer of Ub to an amino acid group of the modified protein. This process causes an isopeptide bond between Ub and lysine through mono-ubiquitylation. The monoubiquitinated protein is then degraded by lysosome [160]. The nature and structure of polyubiquitinated and mono-ubiquitinated proteins are listed in Table 1

Degradation of the HNE-modified proteins occurs via two pathways: the lysosomal pathway and the UPP [137, 138]. The UPP and lysosomal pathways control the degradation of intracellular modified proteins, while extracellular modified proteins are only degraded by the lysosomal pathway [139, 140]. UPP is responsible for the degradation of abnormal cytosolic and nuclear proteins in eukaryotic cells: the 26S proteasome plays a key role in the degradation of proteins [141–143]. The 26S proteasome comprises the 20S proteasome and 19S regulatory particles [144, 145]. The 20S proteasome has been identified as the catalytic core [146, 147]. Studies have shown that the 20S proteasome degrades oxidised proteins without ATP hydrolysis and conjugation of ubiquitin [148–150]. For the ubiquitination of proteins, the 26S proteasome requires ATP hydrolysis and the conjugation of ubiquitin to modify the protein target (Fig. 4) [151, 152]. The aggregation of HNE-modified proteins may lead to cellular dysfunction and cellular aging; in short, the clearance of HNE-modified proteins is crucial to proper cell functioning [133, 153, 154].

**Table 1:** The nature and structure of polyubiquitinated and mono-ubiquitinated proteins

Comparison list	Mono-ubiquitinated proteins	Polyubiquitinated proteins
Formation	Ubiquitin (Ub) forms a thioester to interact with E1; Ub is transferred from E1 to E2; E3 interacts with Ub-charged E2, resulting in an isopeptide bond between Ub and lysine	Ub forms a thioester to interact with E1; Ub is transferred from E1 to E2; E3 interacts with E2, which enables the conjugation between lysines and Ub chain, leading to further cycles of ubiquitination
Protein structure	Less structural disorder	More structural disorder
Ub-site structure in yeasts	More structure disorder	Less structure disorder
Ub-site structure in humans	Less structure disorder	More structure disorder

E1, E2, and E3 are Ub-activating enzyme, Ub-conjugating enzyme, and ubiquitin ligase, respectively. For a comparison, see Ref [161, 162]

Although HNE-modified proteins can be cleared via UPP, this is insignificant for studies of biological reactions because, in some cases, membrane receptors facilitate their degradation via the lysosomal pathway [137, 155]. This finding indicates that both the UPP and the lysosomal pathway are involved in the degradation of HNE-modified proteins (Fig. 4) [23]. In the lysosomal pathway, the HNE-modified proteins are transferred as protein substrates to the receptor of the lysosomal membrane [156, 157]. Consequently, the protein substrates are transformed into the lysosomal lumen and degraded. One study has shown that inhibiting the ubiquitin–proteasome system or the lysosomal proteolytic system alone, leads to a partial decrease in the degradation of the methylglyoxal (MGO)-modified proteins. In contrast, inhibition of both shows a significant aggregation of MGO-modified proteins [158].

AβOs, but not the monomers, impair the 26S proteasome activity (proteasomal activity) [163]. Interactions between the AβOs and the 20S proteasome may lead to the impairment of the proteasomal activity [164]. Since AβO binding to the 20S proteasome may occur in neurodegenerative diseases [165], regulating the proteasome gate may provide a strategy to protect the proteasomal activity against AβO conjugation [166].

**Fig. 5:**
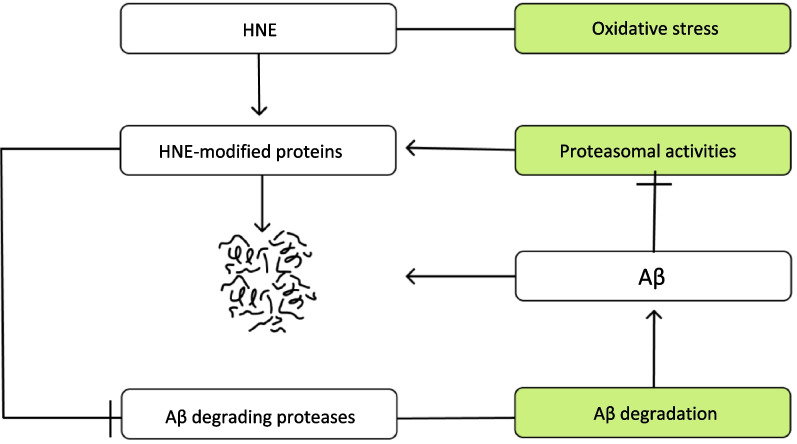
Schematic diagram illustrating proteasome activities, Aβ degradation, and oxidative stress which causes the Aβ degrading proteases to malfunction. (1) Aβ-degrading proteases (IDE and NEP) degrade monomeric and oligomeric forms of Aβ through Aβ degradation [78]. (2) Oxidative stress modifies the Aβ-degrading proteases via HNE, a product of lipid peroxidation. (3) The HNE modification of proteins, caused by oxidative stress, impairs the activities of Aβ-degrading proteases. (4) Proteasome degrades the HNE-modified proteins via the ubiquitin–proteasome and lysosomal pathways. (5) However, Aβ oligomers can inhibit all of the proteasome activities by inhibiting the 19S proteasome, resulting in the impairment of proteasome activity

Although proteasomal activity contributes to Aβ clearance (through degradation of the HNE-modified proteins), it may also be impaired by conjugation of the AβOs (Fig. 5). Therefore, the proteasomal activity and the AβOs can interact with each other.

## Strategies for alleviation of oxidative stress

In the above sections, we have explored the strong hypothesis that oxidative stress could be the pathogenic operator in the early stage of AD. In the following section, we will discuss potential strategies for alleviating oxidative stress, including the use of synthetic antioxidants, changes in gene expression, and protein design.

### Synthetic antioxidants

Along with increased levels of oxidative damage, decreased antioxidant enzyme activity may cause AD; this process is thought to be due to the resulting oxidative damage to related residues [167]. Thus, enhancement of the antioxidant activity may provide a form of intervention for AD. GSH has been identified as the most prevalent antioxidant in cells of the brain [24, 25]. Studies have shown two effective precursors to GSH: *N*-acetyl-*L*-cysteine (NAC) and γ-glutamyl cysteine ethyl ester (GCEE) [135, 168]. NAC crosses the BBB and provides cysteine for GSH synthesis, thereby increasing GSH levels [25]. Researchers have developed mitochondria-targeted nanocarriers to deliver NAC, and reported that they may halt or slow the degenerative process, which ultimately leads to decreased oxidative damage [169]. GCEE, another precursor which increases GSH synthesis through catalysation, could interact with GSH to easily cross the cell membrane and BBB [25]. This mechanism may provide protection against myocardial dysfunction and mitochondria damage [170].

Although antioxidants play a major role in defending against oxidative stress, the levels of oxidative damage are still greater than the antioxidant defence. Therefore, the antioxidant defence strategies could be integrated into the antioxidant defence network, which could include maintaining conserved mechanisms involving kinases and transcription factors, restricting food intake, increasing exercise, using chemical compounds to increase antioxidant levels by inhibiting reactions catalysed by iron and copper, and promoting lifestyles that reduce oxidative stress [171].

### Changes in gene expression

Changing gene expression levels using gene-based techniques to resist oxidative stress is challenging. Mimicking enhanced antioxidant levels has been proposed as a defensive strategy that could be used to protect cells against oxidative stress. This aim could be achieved by elevating levels of glucose-regulated proteins (e.g., GRP78 and GRP94), and the heat chock protein [56, 172]. To identify genes involved in resistance to oxidative stress, one study performed gene microarray experiments in mammalian cells showing resistance to oxidative stress [173]. Altering certain stress-responsive genes such as *HO*-*1* (heme oxygenase-1), *C-JUN*, and *GADD15* results in elevations of the antioxidant level and GSH level, enabling greater resistance to oxidative stress [174]. An error-prone PCR technique to mutate cAMP receptor protein genes has been used to generate three *E. coli* mutants with improved oxidative stress resistance [175].

### Reducing oxidative stress through protein design

Protein design is a useful technique with potential therapeutic applications, and has been used to reduce oxidative stress via a few techniques, including the development of nanozymes and the creation of protein inhibitors and activators.

Due to their flexible design and economical production costs, nanozymes provide a viable way to study enzymatic and non-enzymatic activities [176]. Nanozymes have been developed to mimic endogenous antioxidants GSH, NAC, and SOD, because these antioxidants scavenge free radicals associated with oxidative damage [177–179]. GSH is an intracellular protective antioxidant that protects cells against oxidative stress in the endoplasmic reticulum. It reduces the non-native disulphide bond and forms the native disulphide bond, and it regenerates other antioxidants such as ascorbate and tocopherols [180, 181]. NAC is a cysteine precursor which contributes to GSH activities by maintaining the synthesis of GSH. Both GSH and NAC function to convert hydrogen peroxide (H_2_O_2_) into water (H_2_O) and dioxygen (O_2_) [182]. This hydrogen peroxide is a result of SOD catalytical conversion of superoxide (O_2_°), one of the free radicals, with the help of cofactors like copper, zinc, and manganese [178, 182]. Some materials have been developed to mimic antioxidants, including metal oxides, carbon-based nanomaterials, and noble metals [183, 184].

For instance, Chen’s group have developed a polyvinylpyrrolidone-modified Prussian blue nanoparticle (PPB) which mimics antioxidant enzyme activities [185]. The goal of PPB is to convert O_2_° to H_2_O_2_ and convert the H_2_O_2_ into H_2_O and O_2_, mimicking GSH, NAC, and SOD. This process ultimately prevents lipid peroxidation and oxidative damage [186]. However, before embracing such technology, we must first understand the biological effects of nanoparticles (their kinetic binding properties and equilibrium), because the inherent shortcomings of nanozymes such as nanoparticles interacting with lipid and DNA impair the expression of the original enzymes [187, 188]. Furthermore, an insufficiency of nanoparticles, and a low ability to interact with the target proteins, are significant drawbacks associated with the development of nanozymes. Both of these limitations will have a negative effect on the treatment [26].

Scientists have found a link between oxidative stress and insulin resistance, suggesting that type 2 diabetes mellitus (T2DM) contributes to AD. This discovery provides an opportunity for pharmacological intervention through the development of protein inhibitors and activators. T2DM, associated with insulin resistance, may cause oxidative stress which contributes to the progression of AD [28, 189]. Studies investigating the mechanisms of the generation of oxidative species have shown that oxidative stress may be directly linked to insulin resistance [190]. Recent studies have identified that insulin resistance is also present in early stages of AD [28, 191]. These studies indicate that alleviating insulin resistance using pharmacological methods may reduce oxidative stress. In pharmaceutical research, scientists have developed various inhibitors and activators to reduce the activities of the Jun NH2-terminal kinase (JNK) and other enzymes that contribute to insulin resistance, such as protein tyrosine phosphatases 1B (PTP1B), fructose-1–6-bisphosphate (FBPase), and glucokinase [192, 193]. Although these inhibitors and activators have been developed to reduce insulin resistance, which may in turn prevent AD, these drugs have side effects; further pharmacodynamics studies are needed to clarify their mechanisms of action, adverse effects, and drug interactions [192, 194–196].

## Strategies of protein design

Well-known strategies of protein design, including directed evolution, rational design, semi-rational design, and de novo design, have been developed in many applications in the hope to resolve issues related to enzyme stability [197]. These strategies require different configurations for implementation, and use different methodologies.

### Directed evolution

Directed evolution is an efficient method for improving the stability and activity of enzymes such as protease subtilisin E [198], cytochrome and GSH transferases [199]. Directed evolution mimics the process of natural selection which includes mutagenesis and selection in vitro [199]. Using this method, researchers have been able to identify selected and mutated residues which improve the catalytic reaction. One experimental study has shown that directed evolution occurs in natural evolution; hence, it can improve the stability and activity of various enzymes [200]. The process of direct evolution begins with the selection of randomly mutated genes from the gene library (> 10,000 clones). Researchers then select and/or screen the gene candidates with the desired function, and isolate the gene candidates, followed by biochemical testing.

### Rational design

Rational design has been developed to improve the thermostability and change the mechanisms of enzymes [201]. Rational design requires information on three-dimensional protein structure and the amino acid sequence as input data and is used for individual mutated gene screening. There are 144,464 protein structures with identified structures available in the Protein Data Bank (PDB) [197]. For enzyme stabilisation, the designed enzymes need to be catalysts for high catalytic activation under mild conditions and require the restricted number of mutations. One study has also developed a strategy to reduce the number of generated variants for rational design, resulting in a reduced screening workload [202]. After protein candidates have been generated by the mutated gene screening process, residue targets may be selected based on substrate selectivity [203]. Unlike directed evolution, in rational design one must understand the interactions between amino acids and protein structures from the beginning of the process.

### Semi-rational design

As rational design requires in-depth knowledge and high-throughput scanning, and directed evolution, based on the randomisation method, can be performed without in-depth knowledge, researchers developed semi-rational design to improve the screening and selection efficiency. This hybrid model is a combination of directed evolution and rational design [204]. Semi-rational design takes advantage of directed evolution and rational design [204]. The smart library has been used to improve randomisation of the directed evolution method by applying in-depth knowledge of the rational design method (knowledge of stability via mutation of enzyme’s residues) to the whole genome [204, 205]. For example, researchers have employed knowledge of Cre recombinase recognition of DNA to improve the randomisation method for rearrangement of DNA [206]. Compared with rational design and directed evolution, the semi-rational design may lead to a higher probability of synergistic mutations [204]. Semi-rational design has its limitations. To obtain the desired protein activities, researchers must still grapple with a complicated design. Also, this approach can only use a limited number of proteins.

### De novo protein design

De novo protein design refers to the inverse protein folding, a process which involves creating a protein from scratch instead of using a known protein structure. The topology of the structural protein design, based on primary sequence, is the basis for de novo design. De novo protein design begins by identifying the scaffold protein. It investigates new functions of proteins, a process depending on knowledge of biomedical and synthetic biology [207]. De novo design consists of two steps: generating protein backbone conformation and detecting combinatorial sidechain packing [208]. De novo design generally uses the Monte Carlo procedure to random peptide fragments—based on the backbone conformation—and calculates the lowest energy needed to stabilise the enzyme [208]. Researchers designed this technique using computational design principles [209], via protein backbones retrieved from the PDB [210]. Rosetta, a well-known computational approach and a software package developed by the Rosetta group, was developed to determine an enzyme’s chemical structure [209]. A remarkable study has applied Rosetta to de novo design (as the protein-design strategy), to improve catalytic efficiency [211]. This study focused on improving enzyme catalysis at the transition state of target enzymes. This study concluded that better binding between the modified enzymes (a result of optimisation in the reactant state) and substrates improves enzyme catalysis, by stabilising the catalytic reaction, in the transition state. Since the metalloproteins (metalloenzymes) are involved in biological and chemical processes in nature—in particular stabilising the catalytic reaction, de novo protein design has been explored for designing and redesigning the metalloproteins [212]. Since the transition states of the catalytic reaction at atomic and electronic levels are crucial to be explored for the protein design, quantum mechanics (QM)/molecular mechanics (MM) calculation method has been applied with the de novo protein design [213]. However, despite this advantage, researchers must still discuss the reaction in the transition state and the reactant state, at the atomic level, to improve the accuracy of QM/MM simulations [211].

### QM/MM

The combination of MM and QM calculations, known as a QM/MM calculation, has benefited charge-density analysis (based on calculation of electrostatic interaction and charge-density distribution) as it enables researchers to explore enzymatic catalysis at atomic and electronic levels [214]. The QM calculation focuses on the region of treated quantum (the QM region) (e.g., bond formation and bond breaking during the chemical reaction). The MM calculation focuses on the surrounding portion of the ligand-receptor interactions (the MM region) (e.g., the interaction between active site residues and ligand residues, explained in Box 1). Due to the limitations associated with time complexity in QM calculations –the order of ten of atoms [215], QM region selection continues to be an active area of research. While computational efficiency has greatly improved over the past decade, handling QM regions with more than 100 atoms [216] is still complicated; more than hundreds of thousands of energy states need to be computed for energy evaluations [217]. Due to this issue, methods that help researchers to select the optimal residue(s) to include in the QM region are essential to extend the QM/MM calculation for large-scale electronic structure simulations [218], e.g., selection of the Aβ and IDE residues for the calculation of electrostatic interaction and charge-density distribution in the transition state of the catalytic activation [219].

**Box 1:** Description of the QM/MM approach

*QM/MM principle*: The QM/MM approach has been applied to molecular dynamic (MD) simulation to simulate and investigate chemical reactions at a molecular level and an atomic level. Two regions of this approach are the QM (inner) and the MM (outer) regions. In catalytic reactions, residues in the substrate are included in the QM region; the remaining system is considered the MM region. QM/MM can be divided into two calculation schemes: the subtractive scheme and the additive scheme
*QM/MM schemes*: There are three steps in the subtractive scheme. The first part of the calculation determines the total amount of force-field energy in the system (*E*_*MM*_), in both the MM region and the QM region. The energy of the QM region is calculated at the level of quantum mechanics (*E*_*QM*_) using Khon-Sham Hamiltonian’s density function theory. Finally, the QM region’s energy is calculated at the level of molecular mechanics (*E*_*MM*_) using the force-field calculation. The subtractive scheme equation is provided below: $E_{QMMM} = E_{MM} \left( {MM_{region} + QM_{region} } \right) + E_{QM} \left( {QM_{region} } \right) - E_{MM} \left( {QM_{region} } \right)$
One of the advantages associated with the subtractive scheme is that no communication is required between the two regions (the QM region and the MM region). However, the polarisation between the QM electron and the MM environment is not considered in the calculation. Furthermore, the subtractive scheme is not flexible and cannot consider chemical change. Unlike the subtractive scheme, calculation of the additive scheme requires coupling between the MM region and the QM region (*E*_*QMMM*_(*MM*_*region*_ + *QM*_*region*_)) instead of (*E*_*MM*_(*QM*_*region*_)). The additive scheme is calculated in the following manner: $EQ_{MMM} = E_{MM} \left( {MM_{region} + QM_{region} } \right) + E_{QM} \left( {QM_{region} } \right) + E_{QMMM} \left( {MM_{region} + QM_{region} } \right)$
Basically, the coupling considers both the force field and the electrostatic potential energies between the QM region and the MM region. The coupling is comprised of bonded and non-bonded energies as shown in the following equation: $E_{QMMM} \left( {MM_{region} + QM_{region} } \right) = E_{QMMM\;bonded} + E_{QMMM\;non\_bonded}$
*The E*_*QMMMbonded*_ is calculated using classical force field theory. The *E*_*QMMMnon_bonded*_ comprises of steric energy (*E*_QMMMsteric_), also calculated using the classical force field theory, and electrostatic potential energy (*E*_QMMMelectrostatic_) and focuses on interaction charges between the
MM region and the QM region. This is calculated using the Schrodinger wave equation: $E_{QMMM\;non\_bonded} = E_{QMMM\;steric} + E_{QMMM\;electrostatic}$
There are three* E*_QMMMelectrostatic_ schemes: mechanical embedding, electrostatic embedding, and polarized embedding. Mechanical embedding calculates the electrostatic charge based on the QM region, without the charge from the MM region. In some methodologies, the electrostatic charge is zero. Electrostatic embedding calculates the electrostatic interaction between the QM and MM regions using the Schrodinger wave function. Finally, polarized embedding considers the polarization between the QM and the MM regions. However, researchers are still working on improving the calculation of the polarized embedding due to the simulations’ ineffective results
*QM/MM Applications*: Due to differences in the expected results and the number of molecules of interest, speed and accuracy are crucial issues when deciding what QM/MM schemes to use. Semi-empirical (such as AM1, MP3) methods have been used to calculate energy at a high level. These calculations require parameters from empirical data. Ab initio (such as HF, MP2, CCSD), is a method used to calculate energy at a low level. While it is more accurate due to its use of Schrodinger’s equation (instead of parameters from empirical data), it has a high computational cost. This limitation means that the ab initio method may not be suitable for computing an entire system of catalytic reactions. The density functional theory (DFT) method was developed to lower computational costs: it reduces the dimensionality of the calculation problem. The figure below provides a comparison of these methods based on their accuracy and speed

## Conclusion

In this paper, we have reviewed oxidative stress and production of HNE, the role of HNE adduction and its effects on the proteases (IDE and NEP). These situations occur in the early stage of AD, causing impairment of Aβ clearance [220]. HNE is a by-product of oxidative stress, which occurs as a result of lipid peroxidation [11, 221]. Conjugations between HNE and amino acids of the proteases result in formation of HNE-NEP and HNE-IDE adducts, known as HNE modification [222].

As a natural mechanism, the reduction of oxidative stress is vital to cellular homeostasis. The modified HNE (HNE-IDE and HNE-NEP adducts) can be degraded in two pathways: the UPP  and the lysosomal pathway. While the UPP needs the 20S  proteasome to degrade the protein adducts without ATP hydrolysis, the lysosomal pathway requires ATP hydrolysis and the 26S  proteasome to degrade the protein adducts [149]. Researchers have designed defensive strategies to reduce oxidative stress by exploiting antioxidant expression, e.g., increasing the activity of transcription factors involved in enhancing the antioxidant expression and using nanozymes to enzymatically mimic antioxidant activity [8, 26]. Nanozymes have the advantages of low cost associated with nanomaterials and multifunctionality [26]. However, using such technology would impair the natural enzymes [223]. To yield a more efficient result, nanozyme design should be developed using the concept of protein engineering, to improve their biocompatibility [26]. Since insulin resistance may be a cause of impaired free fatty acid (FFA) degradation, and the aggregation of FFA may lead to oxidative stress, the inhibition of enzymes contributing to insulin resistance and activation may support resistance to oxidative stress and offer a form of early intervention [224, 225]. Researchers have recently developed JNK inhibitors, PTP1B inhibitors, FBPase inhibitors, and glucokinase activators to regulate activities associated with insulin resistance [192–194, 196].

In this paper, we have also described concepts and strategies of protein design and their role in the reduction of oxidative stress. In particular, the QM/MM calculation method is used to calculate charge-density distributions and electrostatic interactions to explore the transition state at atomic and electronic levels. Selection of residues at the ligand-binding domain is a crucial strategy for extending the QM/MM calculation for large-scale electronic structure simulations.

In conclusion, understanding the interactions between HNE and amino acids of proteases may explain how oxidative stress impairs Aβ clearance. Furthermore, transcription factor regulation involved in the enhancement of antioxidant expression and enzymatic mimicking of the antioxidant activities present challenges for protein design in terms of reducing oxidative stress. The QM/MM methods may help improve protein design and contribute to the treatment of AD in early stage.

## Data Availability

Not applicable.

## Abbreviations

Aβ: Amyloid beta
AD: Alzheimer’s disease
APP: Amyloid protein precursor
ATP: Adenosine triphosphate
HNE: 4-Hydroxynonenal
ROS: Reactive oxygen species
RNS: Reactive nitrogen species
GSH: Expression of glutathione
SAD: Sporadic Alzheimer’s disease
aMCI: Amnestic mild cognitive impairment
Lys: Lysin
His: Histidine
Cys: Cysteine
E3: Ubiquitin ligase enzyme3
Ub: Ubiquitin chain
MGO: Metabolic α-dicarbonyl compound methylglyoxal
NAC: N-acetyl-L-cysteine
GCEE: γ-Glutamyl cysteine ethyl ester
BBB: Blood brain barrier
GRP: Glucose-regulated proteins
SOD: Superoxide dismutase
FFA: Triglycerides produces free fatty acid
JNK: Jun NH2-terminal kinase
PTP1B: Protein tyrosine phosphatases 1B
FBPase: Fructose-1–6-bisphosphate
PDB: Protein data bank
QM/MM: Quantum mechanics and molecular mechanics
IDE: Insulin-degrading enzyme
